# Low-Quality Sensor Data-Based Semi-Supervised Learning for Medical Image Segmentation

**DOI:** 10.3390/s24237799

**Published:** 2024-12-05

**Authors:** Hengfan Li, Xuanbo Xu, Ziheng Liu, Qingfeng Xia, Min Xia

**Affiliations:** 1 Jiangsu Collaborative Innovation Center for Atmospheric Environment and Equipment Technology, Nanjing University of Information Science and Technology, Nanjing 210044, China; 202491000006@nuist.edu.cn; 2 Institute of Systems Science, National University of Singapore, Singapore 119077, Singapore; xuanbo_xu@u.nus.edu; 3 Department of Computer Science, University of Reading, Whiteknights House, Reading RG6 6DH, UK; fp845200@student.reading.ac.uk; 4 School of Automation, Wuxi University, Wuxi 214105, China; xqf@cwxu.edu.cn

**Keywords:** deep learning, semi-supervised, hard region, entropy, medical image sensor

## Abstract

Traditional medical image sensors face multiple challenges. First, these sensors typically rely on large amounts of labeled data, which are time-consuming and costly to obtain. Second, when the data volume and image size are large, traditional sensors have limited computational power, making it difficult to effectively train and infer models. Additionally, traditional sensors have poor generalization ability and struggle to adapt to datasets with different modalities. This paper devises a novel framework, named LSDSL, and deploys it in the sensor. LSDSL utilizes low-quality sensor data for semi-supervised learning in medical image segmentation. in supervised learning, we devise the hard region exploration (hre) module to enhance the model’s comprehension of low-quality pixels in hard regions. in unsupervised learning, we introduce a pseudo-label sharing (ps) module, which allows low-quality pixels in one network to learn from the high-quality pixels in the other networks. our model outperforms other semi-supervised methods on the datasets of two different modalities (CT and MRI) in medical image sensors, achieving superior inference speed and segmentation accuracy.

## 1. Introduction

In the field of medical imaging, the rapid development of image segmentation technology has provided significant support for the application of sensors [1,2]. Medical image segmentation aims to separate important structures from the background in images, thereby accurately extracting key biological information. This technology brings substantial benefits to the performance enhancement of medical sensors, particularly in diagnostic and therapeutic applications [3].

Through image segmentation, sensors can more effectively identify pathological regions, thereby improving the accuracy and reliability of diagnoses [4]. For example, in tumor detection, precise image segmentation helps sensors clearly delineate tumor boundaries, which not only improves tumor size measurements but also provides more reliable treatment options [5].

In addition, by incorporating advanced deep learning methods such as semi-supervised learning, image segmentation can reduce the reliance on large amounts of labeled data, allowing sensors to adapt more effectively to different clinical environments [6,7,8,9,10,11,12,13]. This advantage is particularly beneficial in resource-limited settings, enabling timely and accurate decision support for physicians. By integrating image segmentation with sensor data, clinicians can obtain vital pathological information in real time, thereby enhancing diagnostic efficiency and improving patient treatment outcomes.

**Figure 1:**
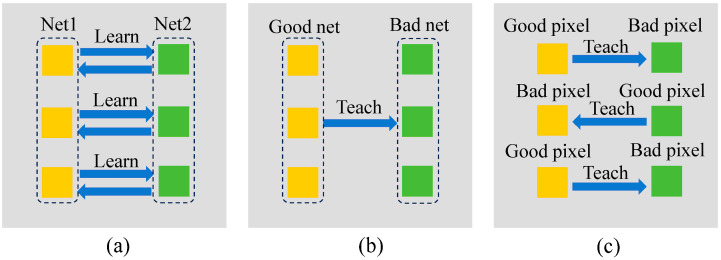
Examples of multiple networks learning from each other in unsupervised learning: (**a**) Two networks learning from each other, (**b**) The good network guides the bad network, (**c**) The good pixels guide the bad pixels.

Semi-supervised medical image segmentation methods often use multi-network structures to learn from each other [14,15]. Figure 1a shows two networks learning from each other, and Figure 1b demonstrates a high-performance network imparting knowledge to a lower-performance network. These approaches focus solely on achieving overall network consistency, neglecting the potential for low-quality pixels to learn from high-quality pixels. We believe these low-quality pixels that are prone to negative effects contain rich feature information. Figure 1c illustrates our pixel learning approach, where high-quality pixels guide their low-quality counterparts, while high-quality pixels are not influenced by low-quality ones. This ensures that the network effectively learns the features of high-quality pixels without being negatively impacted by the features of low-quality pixels.

So, to obtain segmented images with high evaluation metrics and excellent segmentation results, it is essential to ensure that the model pays enough attention to low-quality pixels during training and derives a positive impact from these pixels. We propose a new semi-supervised medical image segmentation method, named LSDSL. Our method comprises two modules: the HRE and PS modules, utilized for supervised and unsupervised learning, respectively. In the supervised learning, the HRE module employs ground truth as a reference to identify regions where the network deems pixel anomalies, designating them as hard regions. The model reinforces its understanding of these difficult areas to improve performance. In unsupervised learning, when two networks predict the same pixel, they produce two different entropies. The pixels with low entropy are considered high-quality sensor image pixels, while those with high entropy are regarded as low-quality sensor image pixels. The PS module encourages the low-quality pixels in both networks to learn from the high-quality pixels, thereby improving performance. Finally, we implement the trained model on the medical image sensor to achieve medical image segmentation. The design of LSDSL aims to address numerous challenges faced by traditional medical image sensors, such as their reliance on large amounts of labeled data, limited computational capabilities, and poor generalization. LSDSL is designed to enhance the utilization of both labeled and unlabeled data, effectively leveraging low-quality data that other methods have not utilized to extract richer semantic information. It can be deployed on medical image sensors for real-time image processing, and presents outstanding performance across datasets of different modalities.

Our main works are summarized as follows:

(1) We introduce a novel medical image segmentation method, LSDSL, to more precisely identify and understand low-quality labels.
(2) We propose an HRE module to make the model more focused on the correct understanding of hard regions in labeled data.
(3) We propose a PS module to enable the utilization of low-quality pixels to their maximum positive impact.
(4) Our model demonstrates the best performance on the medical image sensor.

## 2. Related Work

### 2.1. Semi-Supervised Medical Image Segmentation

Currently, there exist numerous commendable semi-supervised methods demonstrating strong performance through diverse techniques. CAML [16] utilizes labeled data to guide unlabeled data, enhancing the extraction of feature information. MTNet [17] combines an attention mechanism with an uncertainty minimization strategy to effectively leverage unlabeled images. BCP [18] enables the model to acquire generic semantic information while learning from unlabeled data. DCPA [19] integrates pseudo-labeling, consistency regularization, and data augmentation. CPR [20] designs a context-similarity learning module to learn contextual relationships and utilized these relationships to correct pseudo-labels. UG-MCL [21] combines intra-task consistency learning and inter-task regularization to exploit geometric shape information. LGDA [22] proposes a local–global pseudo-label correction method for source-free domain-adaptive medical image segmentation. MCF [23] explores network bias correction. SNSCL [24] designs a noise-tolerant supervised contrast learning loss for correcting noisy labels. PH-Net [25] masks out hard regions while randomly cropping other regions for data augmentation, employing a contrastive learning method to improve the segmentation performance of hard regions. However, these methods often overlook network interactions and the potential negative impact of incorporating low-quality data during training.

### 2.2. Pseudo-Labeling Method

Semi-supervised semantic segmentation methods often employ pseudo-labeling techniques to acquire reliable feature information, thereby enhancing performance. Fixmatch [26] identifies high-quality pseudo-labels by applying a fixed threshold. Subsequently, UA-MT [14] uses uncertainty estimation to select more reliable pseudo-labels. UPC [27] combines consistency regularization and pseudo-labels to correct pseudo-labels containing noise by uncertainty. RPG [28] establishes matches between visually similar regions in labeled and unlabeled images to share semantic information. SCP-Net [29] uses unlabeled data to enhance the coherence of pseudo-labels within each class. ACPL [30] proposes an anti-curriculum pseudo-labeling method that improves pseudo-labels accuracy through the precise integration of classifiers. PCL [31] suggests a multi-round correction method and a multi-vote weighting method for pseudo-label correction. DISC [32] selects and corrects noisy labeled data based on the momentum of memorized intensities of each instance in previous rounds. Rankmatch [33] proposes a rank-aware correlation consistency strategy to enhance the model’s generalization performance. CroCT [34] introduces a novel cross-structure and task-cooperative teaching mechanism. CCDC [35] dynamically selects the most representative pixels to form positive and negative pairs, enabling contrastive learning at different training stages. PLMT [36] synergizes the self-training pipeline with pseudo-labeling and consistency regularization techniques. However, none of these methods fully harness the potential of low-quality pseudo-labels.

## 3. Method

### 3.1. Overview

Before presenting our method, we introduce some notations to define the semi-supervised medical image segmentation task. The purpose of this is to describe our method succinctly. We are given the entire dataset $D{=D}_{L}+D_{U}$: $D_{L}$ is the labeled dataset consisting of *N* images, and $D_{U}$ is the unlabeled dataset consisting of *M* images, where *N* ≪ *M*. Next, the two datasets can be formalized as $D_{L}=\{x_{i}^{L},y_{i}^{L}|i\in (1,\ldots ,N)\}$ and $D_{U}=\{x_{i}^{U}|i\in (1,\ldots ,M)\}$, where $x_{i}^{L}$ and $y_{i}^{L}$ denote the images in the labeled data and the corresponding ground truth, respectively, and $x_{i}^{U}$ denotes the images in the unlabeled data, $x_{i}^{L}{,x}_{i}^{U}\in R^{H\times W\times D}$, $y_{i}^{L}\in {\left\{0,1\right\}}^{H\times W\times D}$.

**Figure 2:**
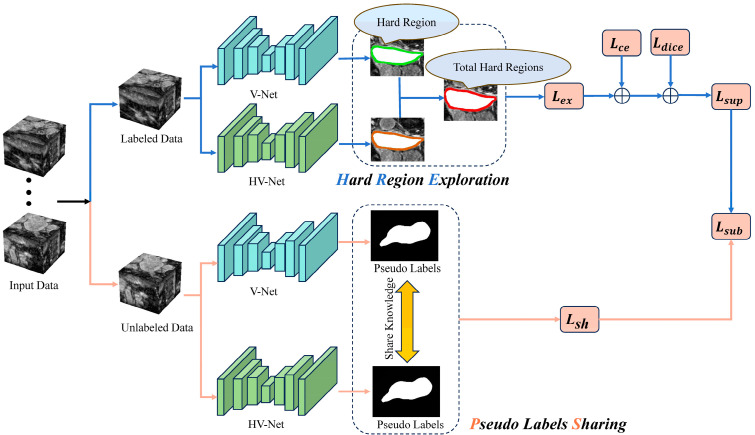
An overview of our proposed LSDSL framework.

Our proposed semi-supervised medical image segmentation method is illustrated in Figure 2. To capture more hard regions, we devise two heterogeneous basic networks that demonstrate comparable performance [23]. Network A adopts the commonly used V-Net architecture, while in network B, we substitute the encoder part of V-Net [37] with a residual structure. This modified architecture is referred to as the Heterogeneous V-Net (HV-Net).

### 3.2. Hard Region Exploration

We observe several abnormal phenomena during the model training process: (1) both networks make consistent predictions, but they do not match the ground truth; (2) pixel labels with higher entropy match the ground truth, while pixel labels with lower entropy do not; (3) there is a significant difference in the entropy of predictions for a given pixel between the two networks. These pixels likely belong to regions with complex features, making it difficult for the model to accurately determine the class, and they are classified as low-quality pixels, which are also considered part of the hard regions. This situation reflects the model’s potential bias in understanding image features in certain regions, leading to predictions that do not align with the ground truth. By identifying these hard regions, the model can focus on learning from areas with rich information but complex features, thereby improving its overall understanding and segmentation capability.

During the supervised learning phase, we design an HRE module that uses labels and entropy as references to explore hard regions in the images. This exploration allows the model to uncover potential and rich feature information in these regions, thereby providing more positive and diverse features to enhance model performance. Thus, we identify three anomalies in network predictions as hard regions:

(1) Two networks simultaneously predict the class of a pixel to be in agreement, but this prediction does not align with the class in the corresponding ground truth, as expressed in the form:

(1) $$M_{1}=I((y_{V}^{L}=y_{H}^{L})\&(y_{V}^{L}\neq y^{L})),$$

(2) These two networks obtain inconsistent entropy for the same pixel. The class of the pixel with higher entropy aligns with the ground truth, while the class of the pixel with lower entropy is inconsistent with the ground truth, as expressed in the form:

(2) $$M_{2}=I(\left(E_{V}^{L}<E_{H}^{L}\right)\&\left(y_{V}^{L}\neq y^{L}\right)\&\left(y_{H}^{L}=y^{L}\right)),$$

(3) $$M_{3}=I(\left(E_{V}^{L}>E_{H}^{L}\right)\&\left(y_{H}^{L}\neq y^{L}\right)\&\left(y_{V}^{L}=y^{L}\right)),$$

here, $y_{V}^{L}$ and $y_{H}^{L}$ are the output classes with the maximum probability from the two networks, given the input labeled data.

(3) The entropy difference between the predictions of a given pixel by the two networks is significant, as expressed in the form:

(4) $$M_{4}=I(Abs\left(E_{V}^{U}-E_{H}^{U})>\tau_{1}\right),$$

The region where these three anomalies occur is defined as the hard region in the image. Here, I(·) denotes the indicator function, and $\tau_{1}$ is the bias threshold and is set to 0.3. $E_{V}^{L}$ and $E_{H}^{L}$ represent the entropy of the labeled data input to the V-Net and HV-Net network predictions, computed by Equation (5).

We define $p_{ij}\in R^{C}$ as the *j*-th pixel of the *i*-th image in the output of the network’s softmax probability, where *C* is the number of classes [38]. Entropy is represented as follows:

(5) $$E\left(p_{ij}\right)=-\overset{C-1}{\underset{c=0}{\sum }}p_{ij}\left(c\right)\log p_{ij}\left(c\right),$$

where $p_{ij}(c)$ is the value of $p_{ij}$ at *c*-th dimensions.

The representation of hard regions is as follows:

(6) $$M_{ex}=M_{1}\cup M_{2}\cup M_{3}\cup M_{4},$$

Therefore, we design a hard region exploration loss to enhance the model’s understanding of hard regions:

(7) $$L_{ex}=\frac{\sum M_{ex}{\left\|y_{K}^{L}-y^{L}\right\|}^{2}}{\Sigma M_{ex}},$$

where $y_{K}^{L}$ refers to the output probabilities $y_{V}^{L}$ and $y_{H}^{L}$ in the V-Net and HV-Net, respectively, and $y^{L}$ is the ground truth.

### 3.3. Pseudo-Label Sharing

The PS module is designed based on the different perceptions of the same pixel by two heterogeneous networks. Due to the differences in the structure and parameters of the networks, their predictions for the same pixel may vary. This difference can be leveraged to extract feature information about the pixel. Pixels predicted as low entropy (i.e., considered high-quality) in one network are used as pseudo-labels to guide the learning of corresponding high-entropy (low-quality) pixels in the other network. Therefore, low-quality pixels can acquire useful information from high-quality pixels, improving their feature representation and thereby enhancing the overall performance of the model. To achieve this goal, an entropy difference mask is designed. This mask selects pixels with a certain entropy discrepancy between the two networks by setting a threshold *β* and a bias threshold $\tau_{2}$, thereby identifying which pixels can serve as high-quality pseudo-labels to guide the learning of low-quality pseudo-labels. In this way, the model can extract valuable information from unlabeled data, enabling the learning of low-quality pixels from high-quality ones.

The entropy difference mask is represented as follows:

(8) $$M_{d}=I(\left(E_{A}^{U}>\beta \right)\&(\tau_{2}<Abs\left(E_{V}^{U}-E_{H}^{U}))\right),$$

The purpose of this mask is to identify pseudo-labels with entropy bias between the two networks. Here, *β* is used as a threshold to filter out pixels with very small entropy and is set to 0.2, $\tau_{2}$ is the bias threshold and is set to 0.05, $E_{V}^{U}$ denotes the entropy of predicted pixels by V-Net, and $E_{H}^{U}$ denotes the entropy of predicted pixels by HV-Net. In their respective networks, $E_{A}^{U}$ represents the corresponding entropy. The computation process of $E_{V}^{U}$ and $E_{H}^{U}$ is similar to that in Equation (5).

Additionally, we designate a portion of pixels with very small entropy as high-quality pixels, and they learn from each other. The mask is set as follows:

(9) $$M_{h}=I\left(0<E_{A}^{U}<\beta \right),$$

The pseudo-label sharing loss $L_{sh}$ is an unsupervised loss, calculated using mean squared error, as follows:

(10) $$L_{sh1}=\frac{\sum M_{d}{\left\|y_{high}^{U}-y_{low}^{U}\right\|}^{2}}{\Sigma M_{d}},$$

(11) $$L_{sh2}=\frac{\sum M_{h}{\left\|y_{V}^{U}-y_{H}^{U}\right\|}^{2}}{\Sigma M_{h}},$$

(12) $$L_{sh}=L_{sh1}+L_{sh2},$$

here, $y_{high}^{U}$ represents pseudo-labels for pixels with relatively high entropy in one network, while $y_{low}^{U}$ represents pseudo-labels for pixels with lower entropy in the other network; they do not require backpropagation during the training process. $y_{V}^{U}$ and $y_{H}^{U}$ represent pseudo-labels for V-Net and HV-Net, respectively.

Finally, the total loss function $L_{sub}$ for each subnetwork is expressed as follows:

(13) $$L_{sup}=L_{ce}+L_{dice}+{\alpha L}_{ex},$$

(14) $$L_{sub}=L_{sup}+{\lambda_{1}L}_{sh},$$

where $L_{sup}$ represents the supervised loss, and $L_{ce}$ and $L_{dice}$ denote commonly used cross-entropy and Dice losses, respectively. *α* is the weight that balances the exploration loss and other supervised losses, and $\lambda_{1}$ is the weight that balances the supervised and unsupervised losses.

## 4. Experiment

### 4.1. Datasets

**Figure 3:**
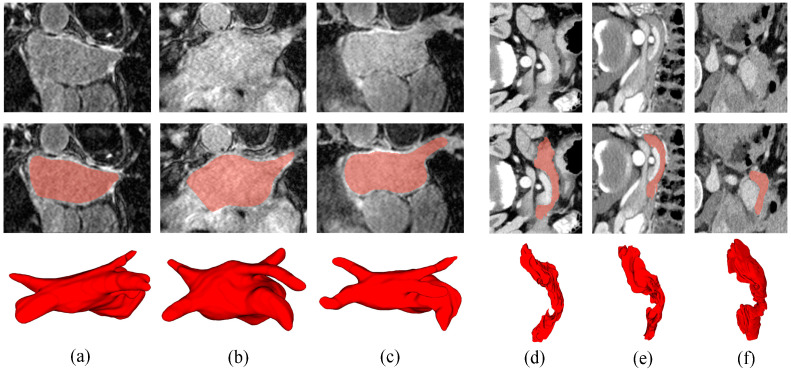
Two-dimensional raw images, Two-dimensional ground truth, and Three-dimensional ground truth of the LA and Pancreas datasets. The first row is the Two-dimensional original image, the second row is the Two-dimensional ground truth, and the third row is the Three-dimensional ground truth. (**a**–**c**) are the images of the left atrium, and (**d**–**f**) are the images of the pancreas.

We conduct evaluations on the Left Atrium (LA) and Pancreas-NIH Segmentation datasets. The first dataset is the LA dataset [39], which comprises 100 imaging scans. This dataset comprises 100 3D gadolinium-enhanced MR imaging volumes, each with corresponding ground truth and an isotropic resolution of 0.625 × 0.625 × 0.625 mm. Figure 3a–c shows the 2D raw images, 2D ground truth, and 3D ground truth of the LA dataset. Medical experts can select images suitable for annotation from a large set of cardiac MRI images. These images need to have sufficient quality and representativeness to accurately reflect the morphology of the patient’s left atrium. The experts use specialized medical image annotation software to carefully outline the boundaries of the left atrium on the images. After completing the annotations, a quality check is usually performed to ensure the accuracy and consistency of the annotations. Multiple experts may annotate the same set of images, and their results are compared. Discrepancies are discussed and corrected, and the annotated image data along with the corresponding annotation information are organized and stored, ultimately forming a complete ground truth dataset of the left atrium. To ensure a fair comparison in experiments, the settings for evaluating our model on this dataset are consistent with other methods [16]. Specifically, we use 80 training volumes and 20 testing volumes. During training, the input volumes are randomly cropped to 112 × 112 × 80 and subjected to flipping as a data augmentation technique.

The second dataset is the Pancreas-NIH dataset [40], containing 82 imaging scans. To ensure a fair comparison in experiments, the settings for evaluating our model on this dataset are consistent with other methods, with a uniform configuration of 62 training volumes and 20 testing volumes.

The in-plane resolution of the CT scan images is fixed at 512 × 512, and the interslice spacing varies between 1.5 and 2.5 mm. Figure 3d–f shows the 2D raw images, 2D ground truth, and 3D ground truth of the Pancreas dataset. On pancreatic CT images, doctors use specialized annotation tools to precisely outline the contours of the pancreas based on its morphology, density, and other features, creating a ground truth. To improve the reliability and accuracy of the ground truth, multiple experts may be invited to annotate the same set of medical images, followed by discussions and negotiations to reach a consensus on the annotations. For disputed regions, further analysis and research are conducted until the final ground truth is determined. During data preprocessing, voxel values are clipped to the range of [−125, 275] Hounsfield Units (HU), and the data are further resampled to an isotropic resolution of 1.0 × 1.0 × 1.0 mm. In the training phase, volumes are randomly cropped to 96 × 96 × 96 before being input into the model. Pancreatic CT volumes, compared to left atrium MRI volumes, exhibit a more complex background. The pancreas, located deep in the abdomen, undergoes significant morphological variations, and its boundaries are often indistinct. Therefore, pancreas segmentation is more challenging than left atrium segmentation due to these factors.

### 4.2. Implementation Details

The training process involves 6000 iterations using the Adam optimizer. We augment the data by applying rotation and flip operations to the images. We conduct the experiments using Python’s PyTorch framework on an RTX 3080 GPU with 12 GB of memory, sourced from NVIDIA, based in Santa Clara, CA, USA. To ensure the objectivity and fairness of comparative experiments while avoiding excessive computational costs, we execute all experiments three times on the same machine with a set of random seeds, presenting the mean and standard deviation of the final iteration results [16]. Regarding framework optimization, we employ the SGD optimizer for model training, setting the initial learning rate to 0.01 and dividing it by 10 every 2500 steps. The batch size is set to 4, containing two labeled patches and two unlabeled patches. To enhance the model’s robustness, we utilize a Gaussian warming-up function $\lambda \left(t\right)=0.1*{\mathrm{e}}^{-5\left(1-t∕t_{max}\right)}$ during the experiment to balance supervised and unsupervised losses, where *t* denotes the current iteration, and $t_{max}$ is the maximum iteration count.

For the quantitative analysis of the experimental results, we utilize four metrics to assess the segmentation performance: Dice coefficient (Dice), Jaccard index (Jaccard), 95% Hausdorff Distance (95HD), and Average Surface Distance (ASD) [27].

### 4.3. Performance on the LA Dataset

In our comparative analysis, we compare LSDSL with other state-of-the-art methods on the LA dataset, including MCF [23], CDMA [17], Co-BioNet [41], MLRP [42], and CAML [16]. We perform experiments on V-Net and HV-Net under fully supervised settings, evaluating the baseline’s performance on the LA dataset trained with 100% and 5% labeled data to establish the upper and lower bounds for the segmentation task.

As shown in Table 1, with only 20% labeled data, LSDSL achieve notable enhancements: Dice increases from 85.97% to 91.25%, Jaccard increases from 75.96% to 83.58%, 95HD decreases from 16.55% to 5.31%, and ASD decreases from 4.87% to 1.59%. Additionally, using only 5% and 10% labeled data, LSDSL also outperforms the other methods in terms of performance.

**Figure 4:**
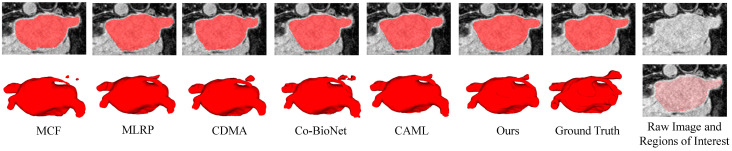
Two-dimensional and 3D visual segmentation results of different semi-supervised methods in the LA dataset with 20% labeled data. Raw image and Regions of Interest show the areas that the model needs to segment in the image.

Figure 4 presents 3D visualizations of the segmentation results for all compared methods alongside their corresponding ground truth. Moreover, LSDSL maintains a lower standard deviation, indicating excellent stability in the designed model.

We test the memory usage and training time of different models on the LA dataset, with the number of training epochs set to 6000. The results are shown in Table 2. Since the network structure of LSDSL is the same as that of MCF, its memory usage and training time are similar. However, compared to other state-of-the-art methods, LSDSL requires fewer computational resources. It can be trained on a 12 GB GPU while achieving higher performance metrics.

### 4.4. Performance on the Pancreas Dataset

**Figure 5:**
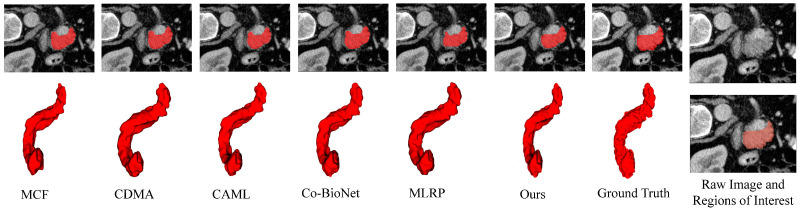
Two-dimensional and 3D visual segmentation results of different semi-supervised methods on the Pancreas dataset with 20% labeled data. Raw image and Regions of Interest show the areas that the model needs to segment in the image.

We compare LSDSL with other methods using 20% labeled data on the Pancreas dataset. The performance results of the baseline network trained on 100% and 5% labeled data for the Pancreas dataset are considered the upper and lower bounds for the task. As shown in Table 3, LSDSL outperforms other methods across all evaluation metrics. When trained with only 20% labeled data compared to the lower bound, LSDSL demonstrates a significant improvement, Dice increases from 64.09% to 77.34%, Jaccard increases from 48.12% to 63.73%, 95HD decreases from 19.02% to 10.93%, and ASD decreases from 4.92% to 2.05%. This substantial performance boost indicates that LSDSL effectively overcomes challenges such as significant morphological variations and the unclear boundaries present in the Pancreas dataset. Figure 5 visualizes the segmentation results of different methods on the Pancreas dataset. Compared to other methods, LSDSL achieves more accurate segmentation.

We conduct statistical tests using LSDSL and other methods on two datasets [43]. The results of the statistical tests demonstrate that the *p*-values for LSDSL compared to the other methods are all less than 0.05, clearly indicating the superior performance of LSDSL.

### 4.5. Effects of Different Components

Table 4 presents the results of the ablation study. In the ablation study, we set (V-Net+HV-Net) as baselines. Subsequently, we incorporate the HRE and PS modules in different combinations to observe the impact of each module on model performance. When the HRE or PS module is added, there is a significant improvement in baseline performance.

With both HRE and PS modules added to the baseline, Dice increases by 4.81%, Jaccard increases by 7.57%, 95HD decreases by 10.47%, and ASD decreases by 4.51%. In supervised learning, the model with the assistance of the HRE module gains a clearer understanding of hard regions in images while minimizing the negative impact of the complex structures of the segmentation target during training. As training progresses, hard regions are no longer difficult to discern. Additionally, in unsupervised learning, the model can leverage the PS module to achieve complementary knowledge. The network imparts the feature information of its high-quality pseudo-labels to the other network.

**Figure 6:**
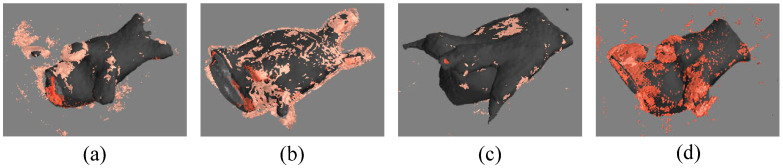
(**a**–**d**) show the low-quality pixels in the left atrium images.

As shown in Table 5, we also calculate the number of low-quality pixels captured by the HRE and PS modules during model training. The model effectively learns from these pixels, leading to a better understanding of the segmentation target. As shown in Figure 6, we highlight the low-quality pixel regions on the segmentation target. It is clear that low-quality pixels are mostly present in hard-to-segment areas.

### 4.6. Effects of HRE Module

After integrating the HRE module into both V-Net and HV-Net, LSDSL exhibits a 10.74% reduction in 95HD and a 2.93% decrease in ASD compared to V-Net. Similarly, compared to HV-Net, LSDSL demonstrates a 10.71% decrease in 95HD and a 2.64% reduction in ASD. The notable decrease in 95HD and ASD affirms the effectiveness of the HRE module in compelling the model to explore hard regions.

**Figure 7:**
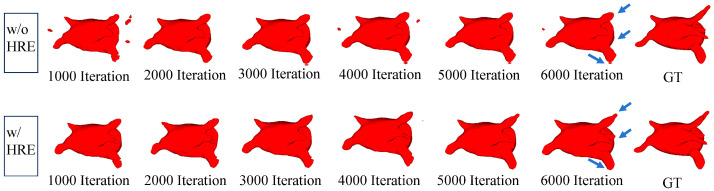
The first and second rows show the segmentation results of LSDSL every 1000 iterations with and without the HRE module, respectively, along with comparisons to the ground truth.

Figure 7 illustrates the positive impact of the HRE module on segmentation results. In the first row without the HRE module, additional noise is present in the segmentation target, and precise segmentation in hard regions like fine branches is compromised. The second row distinctly showcases the favorable influence of the HRE module, indicating that the segmentation target in fine branches closely resembles the ground truth after incorporating the HRE module. This strongly suggests that the HRE module enhances the model’s understanding of hard regions within the segmentation target, resulting in contours that closely align with the ground truth.

### 4.7. Effects of PS Module

**Figure 8:**
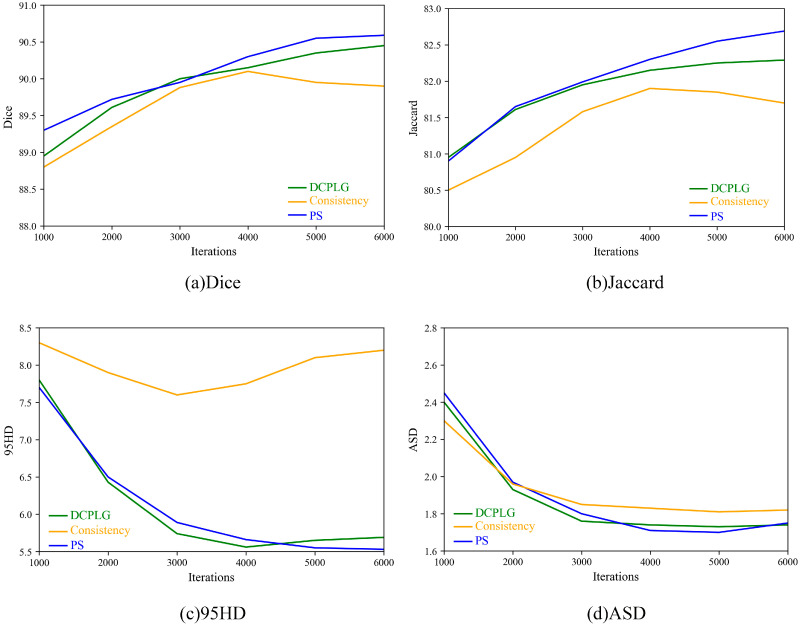
Performance comparison of consistency regularization, MCF’s DCPLG, and our PS module on the LA dataset with 20% labeled data.

The PS module requires simultaneous updates of both networks during training to be effective. Therefore, we demonstrate the module’s effectiveness only when training the two networks simultaneously. After incorporating the PS module into the baseline, LSDSL exhibits an increase of 4.38% in Dice and 6.10% in Jaccard. Figure 8 compares consistency regularization, the DCPLG module in MCF, and our PS module on the 20% labeled LA dataset, highlighting that the PS module demonstrates a more stable performance improvement compared to other network learning methods. This signifies that the PS module directs low-quality pseudo-labels to learn positive features from high-quality pseudo-labels, and the incorporation of low-quality pseudo-labels in training has a beneficial impact.

### 4.8. Performance of Using Different Percentages of Thresholds

**Figure 9:**
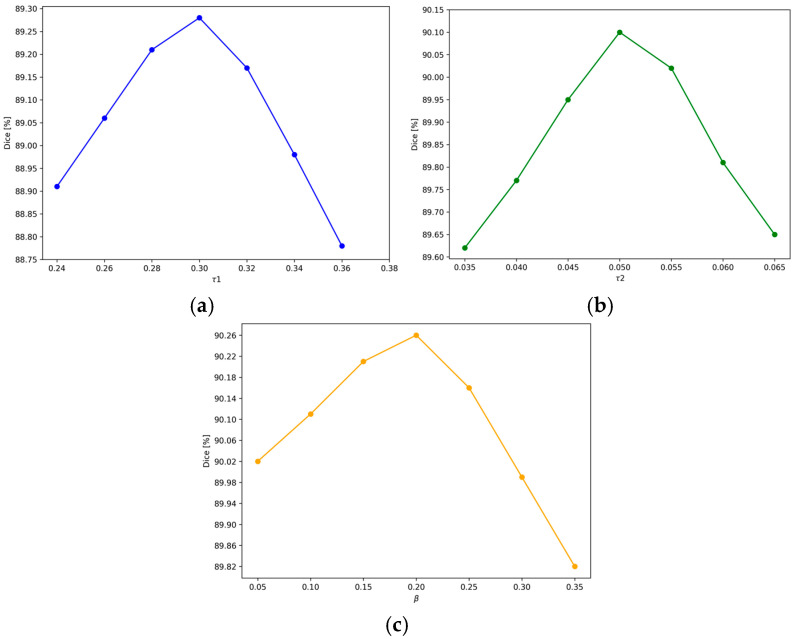
Dice performance with different percentages of thresholds on LA dataset. (**a**–**c**) The effect of $\tau_{1}$, $\tau_{2}$ and *β*, respectively.

As shown in Figure 9, we investigate the influence of $\tau_{1}$, $\tau_{2}$, and *β* on the performance of LSDSL. In Figure 9a–c, the Dice increases correspondingly with the increase of $\tau_{1}$, $\tau_{2}$ and *β*. Dice reaches its peak values at 89.28%, 90.30%, and 90.26%, respectively, and further increases in these values lead to a gradual decrease in Dice.

### 4.9. Performance on the ACDC Dataset

As shown in Table 6, LSDSL is tested on the 2D ACDC dataset [44] and achieves the best performance, demonstrating its effectiveness in 2D datasets and its strong generalization capabilities.

### 4.10. Model Evaluation on Medical Image Sensor

**Figure 10:**
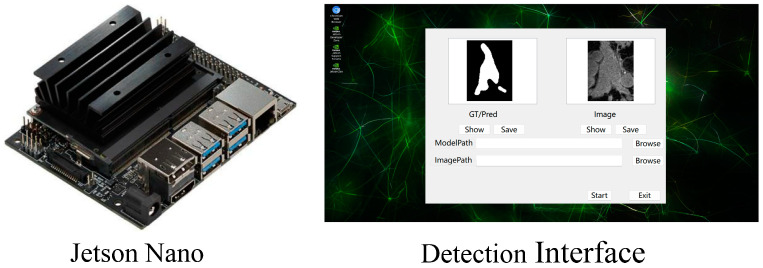
Evaluation on medical image sensor.

We compare the performance of LSDSL with other semi-supervised models on the medical image sensor, as shown in Figure 10, which consists of a Jetson Nano development board and a display [45]. We conduct experiments using the LA dataset with 20% labeled data. The experimental results in Table 7 demonstrate that LSDSL achieves the highest average segmentation metrics and the fastest inference time per image using 20 medical images for inference on the sensor.

### 4.11. Effects of Data Preprocessing on Performance

Cropping and rotating images are used as data augmentation operations before images are input into the model. We conduct ablation experiments to observe the impact of these data augmentation operations on the model’s performance.

In the ablation experiments, we set three different cropping sizes. Since the number of input channels for the network is set to 16, all cropping sizes are multiples of 16. The cropping size of ‘112 × 112 × 80’ is used before model training, which matches the cropping setting in other semi-supervised methods [16]. Since some images have dimensions smaller than 128 or 96 before cropping, we do not conduct further experiments with larger cropping sizes. The results are shown in Table 8. The performance shown by the size of ‘112 × 112 × 80’ is the best. The reason is that when the image size is cropped to 64 or 48, the target segmentation area can be cropped, which leads to the inability of the model to learn the segmentation target effectively.

Additionally, we apply data augmentation operations such as random flip, 90-degree flip, 180-degree flip, and 270-degree flip to test their impact on the model’s performance and its rotational agnosticism. The results are shown in Table 9. When the image undergoes a 180-degree rotation, the model achieves the highest performance. For other rotation angles, the performance slightly decreases, but it still shows higher performance compared to no rotation, indicating that applying rotation operations can improve model performance. We conduct an additional experiment where the images are input into different models for training without applying any rotation operations. During the testing phase, we perform inference using randomly rotated images. The experimental results, as shown in Table 10, show that the performance of LSDSL only slightly decreases, while other advanced semi-supervised methods experience significant performance drops. This demonstrates that LSDSL is less negatively affected by image rotation operations. Furthermore, we conclude that the model exhibits rotational agnosticism.

### 4.12. Research Gap

We have identified several limitations in the designed method: (1) Although the method uses two basic networks, the computational load of both networks remains relatively high, which imposes a certain computational burden on the hardware module of the sensor. (2) LSDSL relies on manually set thresholds to improve performance when using low-quality data, and these thresholds need to be adjusted if a different dataset is used. (3) When faced with more complex medical images, LSDSL also struggles to achieve good segmentation results, as it only uses a portion of the low-quality data for learning.

## 5. Conclusions

This paper presents a semi-supervised medical image segmentation framework, named LSDSL, designed for deployment on sensors. The core idea is to utilize two basic networks to design a semi-supervised learning model capable of effectively leveraging low-quality pixels. This model can be deployed on sensors and quickly achieve efficient segmentation results. Consequently, we propose two modules in the model: the HE module utilizes ground truth as a reference to guide the model in enhancing its understanding of hard regions. The PS module enables the improvement of the effectiveness of low-quality pseudo-labels and thereby makes a positive impact on the overall model performance. The segmentation results on two different modalities of benchmark datasets indicate that the proposed model’s segmentation contours closely align with the ground truth contours, especially excelling in hard regions while achieving state-of-the-art performance with the medical image sensor.

Currently, the network parameters of the model we designed are still relatively large, and both segmentation accuracy and speed need further improvement. In future work, we will focus on improving network structure to reduce the model’s parameters while maintaining high segmentation performance, aiming to achieve faster and more efficient medical image segmentation on sensors. At the same time, we will enable the model to leverage more low-quality data to improve performance without being dependent on threshold settings.

## Figures and Tables

**Table 1:** LSDSL is compared with other methods on the LA dataset. The metrics display the mean ± standard deviations for each method using three different random seeds.

Method	Volumes Used		Metrics			
	Labeled	Unlabeled	Dice (%)	Jaccard (%)	95HD (Voxel)	ASD (Voxel)
MCF [23]	4(5%)	76(95%)	83.16±1.87	70.86±2.63	18.66±3.12	6.54±0.72
CDMA [17]	4(5%)	76(95%)	87.13±2.31	77.14±1.98	13.24±2.64	3.55±1.54
Co-BioNet [41]	4(5%)	76(95%)	87.26±1.31	77.32±1.65	10.05±1.84	2.88±0.88
CAML [16]	4(5%)	76(95%)	87.34±1.25	77.65±0.08	9.76±0.92	2.49±0.22
MLRP [42]	4(5%)	76(95%)	87.54±1.71	77.31±2.11	9.67±2.51	2.55±1.64
LSDSL(Ours)	4(5%)	76(95%)	87.66±2.35	78.12±1.13	9.37±1.21	2.22±0.65
MCF [23]	8(10%)	72(90%)	86.88±0.64	77.21±0.61	11.24±1.67	3.64±1.78
CDMA [17]	8(10%)	72(90%)	88.56±1.12	80.89±0.98	11.54±2.21	2.84±2.10
Co-BioNet [41]	8(10%)	72(90%)	89.40±0.98	81.10±0.62	9.38±1.89	2.61±1.98
CAML [16]	8(10%)	72(90%)	89.62±0.20	81.28±0.32	8.76±1.39	2.42±0.17
MLRP [42]	8(10%)	72(90%)	89.68±1.56	81.54±0.77	8.72±2.45	2.24±2.15
LSDSL(Ours)	8(10%)	72(90%)	89.88±0.68	81.66±0.48	8.61±1.97	1.83±0.65
MCF [23]	16(20%)	64(80%)	90.48±0.19	82.76±1.13	6.55±0.33	1.85±0.17
CDMA [17]	16(20%)	64(80%)	90.57±0.33	83.28±0.84	6.08±0.51	1.63±0.21
Co-BioNet [41]	16(20%)	64(80%)	90.65±0.20	83.17±1.18	5.98±0.30	1.71±0.15
CAML [16]	16(20%)	64(80%)	90.78±0.11	83.19±0.97	6.11±0.38	1.68±0.16
MLRP [42]	16(20%)	64(80%)	90.84±0.34	83.40±0.85	5.41±0.22	1.62±0.24
LSDSL (Ours)	16(20%)	64(80%)	91.25±0.13	83.58±0.76	5.31±0.35	1.59±0.29

**Table 2:** Memory usage and training time of different semi-supervised methods.

Method	Metrics	
	Memory (GB)	Time (Hour)
MCF [23]	10.61	2.50
CDMA [17]	16.24	3.40
Co-BioNet [41]	27.62	3.57
CAML [16]	26.49	3.32
MLRP [42]	17.82	3.10
LSDSL (Ours)	11.26	2.51

**Table 3:** Comparison of LSDSL with other methods on the Pancreas dataset. Metrics are displayed as the mean ± standard deviation results for different methods using three different random seeds.

Method	Volumes Used		Metrics			
	Labeled	Unlabeled	Dice (%)	Jaccard (%)	95HD (Voxel)	ASD (Voxel)
MCF [23]	3(5%)	59(95%)	50.39±1.48	45.44±1.55	35.49±1.88	13.67±2.22
CDMA [17]	3(5%)	59(95%)	54.59±1.88	48.27±1.89	31.41±1.67	12.44±3.56
Co-BioNet [41]	3(5%)	59(95%)	54.66±2.12	48.48±1.62	29.43±1.01	11.21±4.37
CAML [16]	3(5%)	59(95%)	54.84±1.01	48.67±1.21	29.84±1.48	11.48±2.92
MLRP [42]	3(5%)	59(95%)	55.03±1.45	48.82±1.11	26.31±1.37	11.23±1.44
LSDSL (Ours)	3(5%)	59(95%)	55.12±1.31	49.17±0.91	26.74±1.44	10.98±1.40
MCF [23]	6(10%)	56(90%)	63.89±2.31	53.08±1.46	21.60±1.46	15.34±1.56
CDMA [17]	6(10%)	56(90%)	68.03±1.98	56.87±1.64	18.54±2.15	9.74±2.74
Co-BioNet [41]	6(10%)	56(90%)	68.14±1.64	57.31±2.47	18.37±3.17	8.54±2.41
CAML [16]	6(10%)	56(90%)	69.61±0.51	57.64±1.53	17.73±2.47	5.64±2.36
MLRP [42]	6(10%)	56(90%)	69.88±0.66	58.69±1.45	15.85±2.34	5.77±2.97
LSDSL (Ours)	6(10%)	56(90%)	69.93±0.46	58.87±1.88	15.64±2.21	5.31±1.19
MCF [23]	12(20%)	50(80%)	73.99±1.12	62.17±1.23	11.62±2.67	2.78±1.94
CDMA [17]	12(20%)	50(80%)	76.54±0.87	63.55±0.84	14.48±2.34	2.54±1.71
Co-BioNet [41]	12(20%)	50(80%)	76.85±0.45	63.36±0.99	15.21±2.22	2.07±1.04
CAML [16]	12(20%)	50(80%)	76.88±0.69	63.38±0.65	12.77±1.89	2.96±1.02
MLRP [42]	12(20%)	50(80%)	77.29±0.84	63.66±0.51	11.22±1.78	2.55±1.41
LSDSL (Ours)	12(20%)	50(80%)	77.34±0.32	63.73±0.45	10.83±1.52	2.05±0.90

**Table 4:** Ablation experiment results for different components in LSDSL on the LA dataset. (V-Net+HV-Net) represents the average performance of the two networks.

Method	Module					
	HRE	PS	Dice (%)	Jaccard (%)	95HD (Voxel)	ASD (Voxel)
V-Net+HV-Net			86.44 ± 0.50	76.01 ± 0.87	15.78 ± 3.88	6.10 ± 0.81
V-Net+HV-Net	√		90.68 ± 0.21	81.20 ± 0.40	6.85 ± 0.45	2.24 ± 0.44
V-Net+HV-Net		√	90.82 ± 0.34	82.11 ± 0.31	7.31 ± 0.78	5.76 ± 0.27
V-Net+HV-Net	√	√	91.25 ± 0.13	83.58 ± 0.76	5.31 ± 0.35	1.59 ± 0.29

**Table 5:** Number of low-quality pixels identified by the HRE and PS modules during training.

Epoch	Number of Low-Quality Pixels	
	Sup	Unsup
1000	123,916	130,484
2000	58,243	178,194
3000	45,362	178,258
4000	37,824	131,606
5000	118,800	232,518
6000	37,791	241,031

**Table 6:** Performance comparison of different methods on ACDC dataset.

Method	Volumes Used		Metrics			
	Labeled	Unlabeled	Dice (%)	Jaccard (%)	95HD (Voxel)	ASD (Voxel)
MCF [23]	7(10%)	63(90%)	85.78 ± 1.46	76.11 ± 1.75	8.21 ± 0.88	2.34 ± 1.07
CDMA [17]	7(10%)	63(90%)	88.45±1.77	80.24±1.24	4.61±0.97	1.44±0.54
Co-BioNet [41]	7(10%)	63(90%)	88.61 ± 1.55	80.07 ± 1.84	4.74 ± 0.54	1.34 ± 0.87
CAML [16]	7(10%)	63(90%)	89.01 ± 1.04	80.41 ± 0.55	4.81 ± 1.07	1.57 ± 0.48
MLRP [42]	7(10%)	63(90%)	89.09±0.84	80.55±0.32	4.49±0.46	1.25±0.36
LSDSL (Ours)	7(10%)	63(90%)	89.15 ± 0.27	80.77 ± 0.47	4.31 ± 0.89	1.22 ± 0.61
MCF [23]	14(20%)	56(80%)	87.24 ± 1.88	79.51 ± 1.97	5.28 ± 0.58	1.96 ± 1.03
CDMA [17]	14(20%)	56(80%)	88.97±0.34	80.71±1.24	4.54±0.81	1.55±0.59
Co-BioNet [41]	14(20%)	56(80%)	89.05 ± 0.48	80.65 ± 1.17	4.60 ± 1.34	1.89 ± 0.68
CAML [16]	14(20%)	56(80%)	89.19 ± 0.22	80.79 ± 1.11	5.26 ± 0.97	1.52 ± 0.42
MLRP [42]	14(20%)	56(80%)	89.85±0.17	80.94±0.94	4.22±0.64	1.59±0.46
LSDSL (Ours)	14(20%)	56(80%)	90.03 ± 0.14	81.24 ± 1.05	4.11 ± 0.87	1.50 ± 0.55

**Table 7:** Performance comparison of different methods on medical image sensor.

Method	Metrics	
	Average Dice (%)	Average Inference Time (s)
CAML [16]	90.65	3.36
MLRP [42]	90.75	3.27
Co-BioNet [41]	90.78	3.78
LSDSL (Ours)	90.89	3.16

**Table 8:** Impact of different cropping sizes on performance.

Crop Size	Metrics	
	Dice (%)	95HD (Voxel)
80 × 80 × 48	90.15	9.16
96 × 96 × 64	91.07	6.95
112 × 112 × 80	91.26	5.59

**Table 9:** Impact of different rotation angles on performance.

Rotation Angle	Metrics	
	Dice (%)	95HD (Voxel)
No	90.85	8.45
Random	91.05	6.17
90	91.11	5.98
180	91.26	5.59
270	91.17	5.74

**Table 10:** Evaluating the performance of different models using randomly rotated images. (Rotation option indicates whether rotation is applied during preprocessing).

Method	Rotation	Metrics	
		Dice (%)	95HD (Voxel)
Co-BioNet		83.44	20.03
Co-BioNet	√	90.66	10.61
CAML		81.73	18.15
CAML	√	90.43	11.97
MLRP		86.17	10.21
MLPR	√	90.74	7.64
LSDSL		89.45	8.24
LSDSL	√	90.76	6.13

## Data Availability

The original contributions presented in this study are included in the article material, and further inquiries can be directed to the corresponding author.

## References

[1] Yuan Y., Du Y., Ma Y., Lv H. (2024). DSC-Net: Enhancing Blind Road Semantic Segmentation with Visual Sensor Using a Dual-Branch Swin-CNN Architecture. Sensors.

[2] Wu S., Huang X., Xu R., Yu W., Cheng G. (2024). Impact Load Localization Based on Multi-Scale Feature Fusion Convolutional Neural Network. Sensors.

[3] Cai X., Zhu Y., Liu S., Yu Z., Xu Y. (2024). FastSegFormer: A knowledge distillation-based method for real-time semantic segmentation of surface defects in navel oranges. Comput. Electron. Agric..

[4] Cui P., Bidzikrillah N.A., Xu J., Qin Y. (2024). Application of the Semi-Supervised Learning Approach for Pavement Defect Detection. Sensors.

[5] Fan J., Hua Q., Li X., Wen Z., Yang M. (2022). Biomedical sensor image segmentation algorithm based on improved fully convolutional network. Measurement.

[6] Wei Q., Yu L., Li X., Shao W., Xie C., Xing L., Zhou Y. Consistency-Guided Meta-learning for Bootstrapping Semi-supervised Medical Image Segmentation. Proceedings of the International Conference on Medical Image Computing and Computer-Assisted Intervention.

[7] Li H., Hong X., Huang G., Xu X., Xia Q. (2023). Uncertainty-guided different levels of pseudo-labels for semi-supervised medical image segmentation. IEEE MultiMedia.

[8] Xu Z., Wang Y., Lu D., Luo X., Yan J., Zheng Y., Tong R.K.-Y. (2023). Ambiguity-selective consistency regularization for mean-teacher semi-supervised medical image segmentation. Med. Image Anal..

[9] Wu Y., Li X., Zhou Y. (2024). Uncertainty-aware representation calibration for semi-supervised medical imaging segmentation. Neurocomputing.

[10] Luo X., Chen J., Song T., Wang G. Semi-supervised medical image segmentation through dual-task consistency. Proceedings of the AAAI Conference on Artificial Intelligence.

[11] He A., Li T., Wu Y., Zou K., Fu H. (2024). FRCNet Frequency and Region Consistency for Semi-supervised Medical Image Segmentation. arXiv.

[12] Wen L., Feng Z., Hou Y., Wang, Wu X., Zhou J., Wang Y. DCL-Net: Dual Contrastive Learning Network for Semi-Supervised Multi-Organ Segmentation. Proceedings of the ICASSP 2024—2024 IEEE International Conference on Acoustics, Speech and Signal Processing (ICASSP).

[13] Zhao Y., Lu K., Xue J., Wang S., Lu J. (2023). Semi-Supervised Medical Image Segmentation With Voxel Stability and Reliability Constraints. IEEE J. Biomed. Health Inform..

[14] Yu L., Wang S., Li X., Fu C.-W., Heng P.-A. Uncertainty-aware self-ensembling model for semi-supervised 3D left atrium segmentation. Proceedings of the Medical Image Computing and Computer Assisted Intervention–MICCAI 2019: 22nd International Conference.

[15] Wu Y., Ge Z., Zhang D., Xu M., Zhang L., Xia Y., Cai J. (2022). Mutual consistency learning for semi-supervised medical image segmentation. Med. Image Anal..

[16] Gao S., Zhang Z., Ma J., Li Z., Zhang S. Correlation-Aware Mutual Learning for Semi-supervised Medical Image Segmentation. Proceedings of the International Conference on Medical Image Computing and Computer-Assisted Intervention.

[17] Zhong L., Liao X., Zhang S., Wang G. Semi-supervised Pathological Image Segmentation via Cross Distillation of Multiple Attentions. Proceedings of the International Conference on Medical Image Computing and Computer-Assisted Intervention.

[18] Bai Y., Chen D., Li Q., Shen W., Wang Y. Bidirectional Copy-Paste for Semi-Supervised Medical Image Segmentation. Proceedings of the IEEE/CVF Conference on Computer Vision and Pattern Recognition.

[19] Chen Y., Wang T., Tang H., Zhao L., Zong R., Chen S., Tan T., Zhang X., Tong T. (2023). Dual-Decoder Consistency via Pseudo-Labels Guided Data Augmentation for Semi-Supervised Medical Image Segmentation. arXiv.

[20] Huai Z., Ding X., Li Y., Li X. Context-Aware Pseudo-label Refinement for Source-Free Domain Adaptive Fundus Image Segmentation. Proceedings of the International Conference on Medical Image Computing and Computer-Assisted Intervention.

[21] Zhang Y., Jiao R., Liao Q., Li D., Zhang J. (2023). Uncertainty-guided mutual consistency learning for semi-supervised medical image segmentation. Artif. Intell. Med..

[22] Ye Y., Zhang Z., Tianb C. (2023). Local-Global Pseudo-label Correction for Source-free Domain Adaptive Medical Image Segmentation. arXiv.

[23] Wang Y., Xiao B., Bi X., Li W., Gao X. MCF: Mutual Correction Framework for Semi-Supervised Medical Image Segmentation. Proceedings of the IEEE/CVF Conference on Computer Vision and Pattern Recognition.

[24] Wei Q., Feng L., Sun H., Wang R., Guo C., Yin Y. Fine-grained classification with noisy labels. Proceedings of the IEEE/CVF Conference on Computer Vision and Pattern Recognition.

[25] Jiang S., Wu H., Chen J., Zhang Q., Qin J. PH-Net: Semi-Supervised Breast Lesion Segmentation via Patch-wise Hardness. Proceedings of the IEEE/CVF Conference on Computer Vision and Pattern Recognition.

[26] Sohn K., Berthelot D., Li C.-L., Zhang Z., Carlini N., Cubuk E.D., Kurakin A., Zhang H., Raffel C. (2020). Fixmatch: Simplifying semi-supervised learning with consistency and confidence. Adv. Neural Inf. Process. Syst..

[27] Lu L., Yin M., Fu L., Yang F. (2023). Uncertainty-aware pseudo-label and consistency for semi-supervised medical image segmentation. Biomed. Signal Process. Control..

[28] Seibold C.M., Reiß S., Kleesiek J., Stiefelhagen R. Reference-guided pseudo-label generation for medical semantic segmentation. Proceedings of the AAAI Conference on Artificial Intelligence.

[29] Zhang Z., Ran R., Tian C., Zhou H., Li X., Yang F., Jiao Z. (2023). Self-aware and Cross-sample Prototypical Learning for Semi-supervised Medical Image Segmentation. arXiv.

[30] Liu F., Tian Y., Chen Y., Liu Y., Belagiannis V., Carneiro G. ACPL: Anti-curriculum pseudo-labelling for semi-supervised medical image classification. Proceedings of the IEEE/CVF Conference on Computer Vision and Pattern Recognition.

[31] He Y., Chen W., Liang K., Tan Y., Liang Z., Guo Y. (2023). Pseudo-label Correction and Learning For Semi-Supervised Object Detection. arXiv.

[32] Li Y., Han H., Shan S., Chen X. DISC: Learning from Noisy Labels via Dynamic Instance-Specific Selection and Correction. Proceedings of the IEEE/CVF Conference on Computer Vision and Pattern Recognition.

[33] Mai H., Sun R., Zhang T., Wu F. RankMatch: Exploring the Better Consistency Regularization for Semi-supervised Semantic Segmentation. Proceedings of the IEEE/CVF Conference on Computer Vision and Pattern Recognition.

[34] Zhang F., Liu H., Wang J., Lyu J., Cai Q., Li H., Dong J., Zhang D. (2024). Cross co-teaching for semi-supervised medical image segmentation. Pattern Recognit..

[35] Chen J., Chen C., Huang W., Zhang J., Debattista K., Han J. (2024). Dynamic contrastive learning guided by class confidence and confusion degree for medical image segmentation. Pattern Recognit..

[36] Li B., Xu Y., Wang Y., Li L., Zhang B. (2024). The student-teacher framework guided by self-training and consistency regularization for semi-supervised medical image segmentation. PLoS ONE.

[37] Milletari F., Navab N., Ahmadi S.-A. V-net: Fully convolutional neural networks for volumetric medical image segmentation. Proceedings of the 2016 Fourth International Conference on 3D Vision (3DV).

[38] Wang Y., Wang H., Shen Y., Fei J., Li W., Jin G., Wu L., Zhao R., Le X. Semi-supervised semantic segmentation using unreliable pseudo-labels. Proceedings of the IEEE/CVF Conference on Computer Vision and Pattern Recognition.

[39] Xiong Z., Xia Q., Hu Z., Huang N., Bian C., Zheng Y., Vesal S., Ravikumar N., Maier A., Yang X. (2021). A global benchmark of algorithms for segmenting the left atrium from late gadolinium-enhanced cardiac magnetic resonance imaging. Med. Image Anal..

[40] Roth H.R., Lu L., Farag A., Shin H.-C., Liu J., Turkbey E.B., Summers R.M. Deeporgan: Multi-level deep convolutional networks for automated pancreas segmentation. Proceedings of the Medical Image Computing and Computer-Assisted Intervention--MICCAI 2015: 18th International Conference.

[41] Peiris H., Hayat M., Chen Z., Egan G., Harandi M. (2023). Uncertainty-guided dual-views for semi-supervised volumetric medical image segmentation. Nat. Mach. Intell..

[42] Su J., Luo Z., Lian S., Lin D., Li S. (2024). Mutual learning with reliable pseudo label for semi-supervised medical image segmentation. Med. Image Anal..

[43] Bosma J., Peeters D., Saha A., Saghir Z., Jacobs C., Huisman H. Reproducibility of Training Deep Learning Models for Medical Image Analysis. Proceedings of the Medical Imaging with Deep Learning.

[44] Bernard O., Lalande A., Zotti C., Cervenansky F., Yang X., Heng P.-A., Cetin I., Lekadir K., Camara O., Ballester M.A.G. (2018). Deep learning techniques for automatic MRI cardiac multi-structures segmentation and diagnosis: Is the problem solved?. IEEE Trans. Med. Imaging.

[45] Qureshi M.F., Amin F., Mushtaq Z., Ali M., Haris A.A., Rana A.Y. Real-Time Weed Segmentation in Tobacco Crops Utilizing Deep Learning on a Jetson Nano. Proceedings of the 2024 International Conference on Engineering & Computing Technologies (ICECT).

